# Research on the consequences of employees’ unethical pro-organizational behavior: The moderating role of moral identity

**DOI:** 10.3389/fpsyg.2022.1068606

**Published:** 2022-12-22

**Authors:** Manlu Zhao, Shiyou Qu

**Affiliations:** School of Management, Harbin Institute of Technology, Harbin, Heilongjiang, China

**Keywords:** unethical pro-organizational behavior, pro-environment behavior, guilt, moral identity, COR, pro-social behavior

## Abstract

**Introduction:**

In recent years, employees’ unethical pro-organizational behavior (UPB) has become a social hot issue. This behavior benefits their organization or colleagues while violating core social ethics. Numerous studies have predominately focused on identifying the antecedents and formation mechanisms of UPB. However, only a few studies have focused on the effects and outcomes of UPB. Moreover, guilt triggered by unethical behaviors can motivate individuals to adopt pro-social behaviors, but studies on the effects of UPB on pro-social behavior of actors are rather limited. Therefore, this study explores the underlying relationship between employees’ UPB and their own pro-environmental behavior based on the conservation of resources theory.

**Methods:**

Through collecting data (*N* = 319) from a Chinese online survey company in different time intervals, the theoretical model was tested by the application of Amos 27.0 and SPSS 25.0 for analysis of the data. The CFA, descriptive analysis, hierarchical regression were illustrated in the article.

**Results:**

This study demonstrated that, through emotions of guilt, employees’ UPB is negatively correlated with their own environmental protection act. While this relationship is being examined, moral identity plays this mediating role, which can moderate the indirect relationship between employees’ UPB and their environmental behavior through guilt.

**Discussion:**

The purpose of the research was to identify the influence mechanisms that contribute to employees’ pro-organizational but unethical behavior. With guilt serving as the mediating variable and moral identity serving as the moderating variable, a research model built on the principle of the conservation of resources theory was constructed. This research examines the impact mechanism and boundary conditions of UPB on individual pro-environmental behaviors from the perspective of employees. This paper discusses the theoretical and practical implications of the report’s results.

## Introduction

With the emergence of a large number of business ethics scandals, various unethical behaviors have started taking place in enterprises today. Unethical behaviors refer to behaviors that violate social and moral standards and endanger organizations or others (Bonner et al., 2017). Most of the previous studies have mainly focused on self-interested unethical behaviors (Greenberg, 2002; Treviño et al., 2014). However, in some work scenarios, employees’ unethical behavior may also be intended to benefit entities other than themselves, including their own organization and its members, which is different from self-interested unethical behavior (Umphress et al., 2010). This type of behavior is defined by Umphress as unethical pro-organizational behavior (UPB), which mainly refers to the behavior that promotes the effective functioning of the organization or its members (such as leaders) and violates the core social values, ethics, laws, or regulations. The pro-organizational character of UPB can easily lead managers to ignore or acquiesce to this behavior, but its nature is still unethical, and it can damage the organizational reputation (Umphress et al., 2010). Existing studies have predominately focused on the antecedents and formation mechanism of UPB. There is less exploration of the potential impact and consequences of UPB, especially the effect of this behavior on the employee at the individual level. However, UPB could affect actors’ cognition and subsequent behavior; for instance, employees will experience ambivalence and anxiety after engaging in UPB, which exacerbates employees’ work–life conflict (Liu et al., 2021). Overall, studies on the effect of UPB on individuals need to be further corroborated and improved. First, the moral compensation theory argues that individuals who engage in unethical behavior will behave more ethically in the following period, and the “sinner” will engage in pro-social behavior to compensate for past immoral acts (Zhong et al., 2009; Ding et al., 2016). So, in a new scenario of unethical behavior (UPB), does the moral compensation effect necessarily occur? Will employees adopt pro-social behavior to compensate for their wrongdoings after implementing UPB? What specific type of individuals’ pro-social behavior does UPB affect in the workplace? Second, pro-environmental behavior is considered a special kind of pro-social behavior (De Groot and Steg, 2009), and this green behavior is more common in organizations under the background of China’s implementation of the “dual carbon” strategy and the promotion of green management models by many organizations (Zhang et al., 2018; Sun et al., 2022). Moreover, there are some complex links between unethical behavior and pro-environmental behavior. So, what effect UPB has on individual pro-environmental behavior needs to be urgently explored.

Under the background that China brings “emission peak” and “carbon neutrality” into the overall layout of national ecological civilization construction, a large number of Chinese organizations have established and implemented corresponding environmental protection policies, and managers have begun to encourage pro-environmental behaviors of employees to improve the environmental performance of organizations (Li et al., 2020; Dong et al., 2022; Yuan et al., 2022). Pro-environmental behavior refers to behavior related to environmental sustainability that individuals engage in or lead and are conducive to the sustainable development of the ecosystem (Peng et al., 2020). Relevant research has pointed out that there are complex relationships between ethical or unethical behaviors in organizations and pro-environmental behaviors and changes in the natural environment (Delmas and Burbano, 2011; Gamma et al., 2018; Dong et al., 2022; Larue et al., 2022). For example, due to the hypocrisy effect on consumption choices, enterprise managers will formulate a large number of policies to motivate employees’ ethical behaviors, especially pro-environmental behaviors (Yuan et al., 2022). Paillé et al. (2015) pointed out that the ethical caring behavior among colleagues can promote individuals to voluntarily help deal with environmental problems at work and thus trigger personal pro-environmental behavior. Although these studies revealed the complex relationships between ethical or unethical behaviors and the environmental as well as pro-environmental behavior, our understanding of the psychological mechanisms of how employees’ UPB affects their pro-environmental behaviors is still incomplete, and it is necessary to explore the circumstances where this effect is enhanced or weakened.

The unethical nature of UPB can induce a moral imbalance pressure on the individual, which can make the actor feel guilty (Wang and Xiao, 2018). The feeling of guilt will promote people to generate pro-social intentions (Xu B. et al., 2021), and people relieve psychological pain caused by guilt by implementing compensatory behavior and helping others, that is, guilt can motivate personal pro-social behavior (Nelissen et al., 2013). Onwezen et al. (2013) pointed out that guilt was associated with environmentally friendly behaviors. Pro-environmental behavior is considered a special kind of pro-social behavior (De Groot and Steg, 2009). Moreover, the study has demonstrated that guilt caused by the rumination of individuals’ past negative behaviors can evoke their pro-environmental behaviors (Adams et al., 2020). Therefore, in the workplace, individuals are likely to relieve guilt through pro-environmental behavior. However, some studies have found that individuals’ experienced guilt does not predict their pro-environmental behaviors (Toner et al., 2012). Therefore, research conclusions on the relationship between guilt and pro-environmental behavior are contradictory. Nevertheless, this article argues that the feeling of guilt caused by UPB may inhibit employees’ pro-environment behaviors (PEBs) as, according to the conservation of resources (COR) theory, resources (e.g., time and energy) that individuals have is rather limited (Hobfoll and Lilly, 1993), and they will possibly conserve personal resources. As a negative emotion, guilt will bring a passive repercussion to individuals, which will deplete their abundant psychological resources and decrease the possibility of employees devoting limited resources to extra-role affairs. As a result, guilt caused by employees’ UPB may weaken their participation in PEBs.

Moral identity refers to the degree of individual’s recognition of being a moral person, and it is defined as a self-image organized around some moral traits (Aquino and Reed, 2002). As an individual difference variable (Shao et al., 2008), employees with high levels of moral identity attach importance to their moral image. Conversely, employees with low levels of moral identity pay little attention to moral self-concept. Indeed, the level of cognitive and emotional resources depleted by guilt caused by UPB also varies with individuals’ levels of moral identity, and it is a boundary condition that affects the level of resource consumption.

This research contributes to the existing literature on UPB and PEBs in three ways. First, this study is one of the first to attempt to link UPB with subsequent pro-social behavior of employees (i.e., pro-environmental behavior), which will improve our understanding of the effect mechanism of UPB. Second, the study unfolds the mechanism through which employees’ UPB exerts an effect on their pro-environmental behavior. Drawing on the COR theory, the study proposes to take guilt as a potential mediator to open the “black box” of how UPB inhibits pro-environmental behavior. Finally, through introducing moral identities, the research explores the boundary condition on the indirect relationship between employees’ UPB and their pro-environmental behaviors, and provides empirical evidence to analyze how UPB affects employees’ cognitions, attitudes, and behaviors.

## Theoretical background and hypotheses

### Theoretical background

In the past 33 years, the COR theory has gradually become one of the most commonly used theories in the field of organizational behavior. This theory, first proposed by Hobfoll (1989), was usually used to explore individual behavior and reactions in stressful situations. COR is based on the tenet that people will try to foster, protect, and retain resources that are of actual or potential value to them as well as acquire new resources, and personal resources are loosely defined as objects, states, and conditions (Halbesleben et al., 2014). The primacy of resource loss is a core principle of COR, which means that resource loss is more remarkable and crucial than resource acquisition. This suggests that the impact of psychological damage from resource loss is much greater than the psychological help from resource acquisition (Hobfoll, 2001). Facing the loss of resources, individuals tend to take measures to protect the remaining resources, which reduce the allocation of resources to the individuals’ role in other scenarios (Vohs and Faber, 2007). So, the principle of the primacy of resource loss can help understand the mechanism of UPB affecting individual pro-environmental behavior. First, from the perspective of UPB pro-organizational characteristics, this behavior actually extends the scope of responsibility of individual work requirements, and individuals need to contribute more time, energy, and effort to the organization. While UPB can enable organizations or members to function efficiently, extended role scope and a sense of responsibility can make individuals feel overburdened with work pressure, which will consume a great deal of the individuals’ cognitive and emotional resources. This will lead them to generate resource conservation motivation. As a result, individuals could reduce resource input for pro-social behaviors (e.g., pro-environmental behaviors) and decrease the occurrence of other extra-role behaviors (Bolino and Turnley, 2005). Second, in terms of the unethical nature of UPB, this behavior may harm external stakeholders and have a negative impact on the reputation of the organization (Umphress et al., 2010), which can bring great mental stress and psychological resource consumption to the actors. Moreover, when individuals discover that their work behavior violates the codes of ethics, they would have feelings of guilt and unease, which cause them deplete large amounts of cognitive and emotional resources, resulting in a significant reduction in resources available for other roles and leading to role conflict (Greenbaum et al., 2014).

**FIGURE 1:**
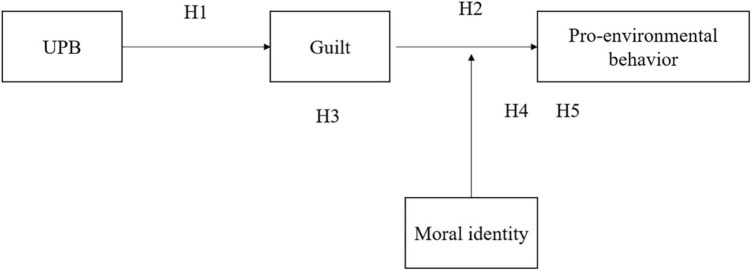
The hypothesized model.

At the individual level, most people aspire to be an ethical person (Aquino and Reed, 2002). However, the implementation of UPB often undermines the ideal moral self, and individuals are likely to experience feelings of guilt and shame. These negative emotions of frustration and anxiety, in turn, deplete the individuals’ emotional resources, and the principle of the salience of resource depletion will force individuals to adopt behaviors that protect existing resources (Halbesleben et al., 2014). Meanwhile, moral identity, as a personal trait, is often used to describe differences among individuals (Shao et al., 2008), and it will significantly affect the level of individual resource consumption. Therefore, in this study, guilt and moral identity are selected as mediating variables and moderating variables separately. Based on the earlier analysis, this study proposes a theoretical model, as illustrated in Figure 1.

### Unethical pro-organizational behavior and guilt

Pro-organizational unethical behaviors refer to behaviors that violate social norms and moral standards in order to help the organization or its members (Lu et al., 2021). Although short-term benefits can be brought to the organization, it will damage the organization’s reputation and personal career due to its unethical nature (Bryant and Merritt, 2021). Umphress and Bingham (2011) pointed out the boundaries of UPB and argued that the behavior has obvious pro-organizational intentions, which was not caused by unconsciousness or carelessness, as well as the starting point of UPB was for the interests of the organization and excluding the satisfaction of personal desires. Existing research mainly focuses on the triggering factors of UPB, which were reflected in three aspects: organizational level, such as ethical climate, organizational competition, and political environment (Kilduff et al., 2016; Valle et al., 2017; Sheedy et al., 2021); leadership factors, such as bottom-line leadership and transformational leadership (Effelsberg et al., 2013; Babalola et al., 2021); personal factors, such as an individual risk of workplace exclusion and perceived organizational support (Thau et al., 2015; Chen et al., 2016). Although there have been some studies on the subsequent effects of UPB (Wang and Xiao, 2018; Liu et al., 2021; Wang Y. et al., 2021), in general, research on the aftereffects of UPB on employees was insufficient.

Previous studies have pointed out that UPB was an unethical behavior (Umphress et al., 2010), which violates social moral norms, while most people strive to become a moral person (Aquino and Reed, 2002). Therefore, as an immoral act, the implementation of the UPB will impair moral self-image (Chen et al., 2022). When employees’ desired ethical self-schema is inconsistent with unethical behavior, it can lead to a negative self-evaluation and passive self-conscious emotions of themselves (Tracy and Robins, 2004). This is extremely likely to induce moral emotions such as guilt (Umphress and Bingham, 2011) because guilt often arises from the negative self-assessment of individuals when they perceive that their actions were unethical (Tang et al., 2020).

Guilt is a universal moral emotion, and it is often associated with an imbalance between the moral self-image and the immoral situation (Livingston and Judge, 2008; Ilies et al., 2013). In some organizational scenarios, unethical work behavior often leads to feelings of guilt (Greenbaum et al., 2019). Shalvi et al. (2015) argued that individuals will trigger moral feelings of guilt after completing unethical behaviors. The nature of UPB is unethical, and this behavior breaks the organizational or social ethical codes (Liu et al., 2021). When individuals realize the immorality of their UPB, they will fall into the ethical whirlpool, and blaming themselves in the process of introspection can induce moral emotions, resulting in the feeling of guilt. Thus, this study proposes the following hypothesis:

**Hypothesis 1:** Unethical pro-organizational behavior will be positively related to guilt.

### Guilt and pro-environmental behavior

As a depressing experience, guilt is closely related to neurosis, anxiety, and depression (Kim et al., 2011), and individuals will experience an emotional process of tension, remorse, and self-blame (Tangney, 2015). When employees experience a mood swing caused by guilt, they need emotion modulation and attention control, as well as behavior guidance, which could greatly deplete their self-regulatory resources (Vohs and Faber, 2007; Zhang et al., 2022). Due to personal resource losses, individuals who have a state of mental fatigue may ultimately show a reduction in their pro-social behaviors (Bolino and Turnley, 2005), and individuals would take measures to protect and maintain their remaining resources (Hobfoll, 2001). So, employees may reduce these pro-social behaviors that require additional resource input at work (Hobfoll, 1989). However, in the context of the current “dual carbon” strategy, employees have to face a particular kind of pro-social behavior, that is, pro-environmental behavior (Sun et al., 2022). Because managers begin to focus on green organizational performance, they pay more attention to the individual green behaviors in the workplace and formulate policies to motivate employees’ pro-environmental behaviors (Zhang et al., 2018). So, employees are most likely to avoid the losses of individual resources by reducing pro-environmental behaviors.

Pro-environment behavior refers to environmental protection behavior that can positively affect the availability of materials or energy and positively alters the structure of ecosystems, and it has a multidimensional structure (Lee et al., 2014; Larson et al., 2015; Coelho et al., 2017). At the individual level, antecedents such as autonomous motivation, values, and emotions can stimulate pro-environmental behaviors (Whitmarsh and O’Neill, 2010; Antonetti and Maklan, 2014; Pauw and Petegem, 2017). At the organizational level, leadership style and organizational climate are external triggers of this behavior (Kim et al., 2018; Xu B. et al., 2021). According to relevant research, emotion plays a considerable role in influencing pro-environmental behaviors. Emotions can express individuals’ true feelings and attitudes about environmental protection, thus affecting their environmental behaviors (Bissing-Olson et al., 2012). Guilt, as a self-conscious emotion, can impact personal cognition and future choices, and empirical research on the effect of guilt on pro-environmental behaviors is relatively extensive (Adams et al., 2020). Moreover, the study showed that there was a positive relationship between guilt and pro-environmental behavior (Elgaaied, 2012). For instance, when considering new purchase decisions, individuals who have experienced guilt will choose sustainable consumption that is conducive to environmental protection (Antonetti and Maklan, 2014). However, some research suggests that there is no association between guilt and pro-environmental behavior (Toner et al., 2012; Bissing-Olson et al., 2016). Although academia has been studying pro-environmental behavior for nearly 40 years, the research results on the relationships between guilt and pro-environmental behavior are still contradictory. In addition, many studies are based on the perspective of moral compensation or social learning (Harth et al., 2013; Wang B. et al., 2021). In other scenarios, research on the effect mechanism of guilt on pro-environmental behavior is still limited.

Based on the COR theory, this study suggests that employees are more likely to feel guilty when they realize the unethical nature of UPB (Umphress and Bingham, 2011), resulting in negative psychology such as fear, anxiety, and depression, which will deplete a large amount of cognitive and emotional resources (Zhang et al., 2022). Although employees have passive emotions after UPB, they still need to maintain a stable emotional and working state in their daily interpersonal interactions, which will further exhaust their psychological resources. Therefore, the guilt caused by UPB will greatly deplete the personal mental resources of employees. Loss salience is a well-established notion of the COR theory, which means that the psychological harm of losing resources is greater than the psychological help of gaining resources, and people are more likely to engage in behaviors that protect their remaining resources and avoid further resource losses (Halbesleben et al., 2014). As a pro-social and extra-role behavior of employees, pro-environmental behavior may aggravate the depletion of individual resources, but the reduction of the occurrence of this behavior will not bring losses to themselves. Therefore, reducing this behavior may be a resource conservation strategy that employees have to adopt. Thus, this study proposes the following hypothesis:

**Hypothesis 2:** Employees’ guilt will be negatively related to their pro-environment behavior.

### The mediator role of guilt

Guilt, as an emotion of self-reflection, originates from the individuals’ awareness of the immorality behind their wrongdoings (Xu et al., 2011). The nature of UPB is immoral, which will result in negative self-assessments of employees. Then, it will bring moral imbalance pressure, and the individuals will have feelings of anxiety and experience guilt (Wang and Xiao, 2018). Therefore, individuals will feel guilty after the implementation of UPB.

According to previous research, individuals have a strong motivation to compensate the people who suffered from their unethical behaviors, thereby keeping their own good moral self-concept (West and Zhong, 2015). A variety of compensatory behaviors can help ease and release negative emotions, including an apology, repentance, and financial compensation for the victim, which also includes compensatory behaviors for unrelated objects, such as voluntary participation in public service activities (Tracy and Robins, 2006; Chrdileli and Kasser, 2018). That is, guilt can promote pro-social behavior. As a type of pro-social behavior, pro-environmental behavior is prevalent in organizations. Employees can relieve the psychological pressure caused by guilt through pro-environmental behavior. Moreover, under appropriate circumstances, guilt can stimulate personal PEB (Rees et al., 2014).

However, our study argues that guilt will negatively affect individuals’ pro-environmental behaviors. Drawing on the COR theory, the resources an individual has are scarce and limited (Hobfoll and Lilly, 1993). When individuals are highly involved in an event, they will invest numerous energy and attention to it, leading to a consumption of psychological resources, so the resources for other roles will be correspondingly reduced (Bolino and Turnley, 2005). The guilt caused by UPB will bring negative emotions and psychological pressure to individuals, and they need to deplete emotional and cognitive resources to alleviate this bad mental state (Zhang et al., 2022). In the face of resource losses, individuals will try to decrease resource investment in other roles and protect the remaining resources (Vohs and Faber, 2007; Halbesleben et al., 2014). Thus, UPB can negatively affect the personal mental state and pro-social behavior through guilt. As a type of extra-role and pro-social behavior, pro-environmental behavior requires employees to undertake more responsibilities beyond the job itself, which needs additional resource investment (Bolino and Grant, 2016; Lu et al., 2017; Xu F. et al., 2021). In short, after UPB depletes some resources of individuals through guilt, the resources invested by employees in pro-environmental behaviors will also decrease accordingly, thereby inhibiting the occurrence of pro-environmental behaviors. Taken together, combining H1 and H2, this study proposes the following hypothesis:

**Hypothesis 3:** Guilt will mediate the relationship between unethical pro-organizational behavior and pro-environment behavior.

### The moderator role of moral identity

Moral identity refers to a self-schema organized around some moral traits, and it is the degree of individual recognition of moral characteristics such as caring, loyalty, kindness, fairness, and justice, which is the integration of self-identity and moral concepts (He and Harris, 2014). Moral identity will significantly affect the realization process of individuals in turning moral beliefs into practical actions (Hardy, 2006). So, moral identity is considered a personal trait, which has been regarded as a moderating variable to describe individual differences (Aquino and Reed, 2002; Shao et al., 2008). Some research studies show that when individuals recall and ruminate the entire process of unethical events, the individuals’ different moral identity standards will directly affect the level of psychological resource losses and subsequent behavioral decision-making (Greenbaum et al., 2014; Joosten et al., 2014). Therefore, this study proposes using moral identity as a moderating variable to explore its contingency effect on individual cognitive paths.

Specifically, employees with low moral identity do not pay much attention to whether their behaviors conform to moral norms and have low moral self-control. Furthermore, they will be more attentive to pro-organizational intent of UPB and benefits to the members in the event, and they are more likely to interpret the whole event and behavior process as a competitive behavior to protect the interests of the organization (Tang et al., 2021). Especially when they believe that such behavior is acquiesced or implied by the organization, they will think the organization would support them in doing so and transfer the responsibility to the organization or managers so as to complete the moral justification of themselves and the cognitive reconstruction of UPB (Ogunfowora et al., 2022). If individuals reinterpret unethical behavior for serving a worthwhile purpose, they will not be plagued by guilt and will not compensate for this behavior (Bandura et al., 1996). So, employees with low moral identity are prone to generate moral disengagement and are less likely to make compensatory behaviors (e.g., pro-environmental behaviors). By contrast, employees with high moral identity will pay more attention to the significance of moral character, and they tend to maintain their own internal moral standards and are not prone to generate moral disengagement (He and Harris, 2014). Even if UPB has already occurred, they are more concerned about the unethical nature and consequences of UPB and are more likely to take compensatory actions to maintain their ideal moral self-image, which can lead to a higher likelihood of employees engaging in pro-environmental behaviors. Thus, this study proposes the following hypothesis:

**Hypothesis 4:** Moral identity will moderate the relationship between guilt and pro-environmental behaviors. Thus, the negative relationship between guilt and pro-environmental behavior will be stronger when the employee has a low level of moral identity.

### Moderated mediation model

In conclusion, guilt plays a mediating role between pro-organizational unethical behavior and pro-environmental behavior. At the same time, guilt and moral identity may have an interaction effect on employees’ pro-environmental behaviors. Based on the earlier analysis, we believe that the influence of employees’ UPB on their own pro-environmental behavior mediated by guilt will be influenced by moral identity. So, this study proposes a moderated mediation model, and it can be seen that moral identity is an important boundary condition that affects this indirect relationship. Employees with less moral values are more likely to use flexible moral self-representation to respond to ethical lapses (Chen et al., 2022), and they may focus more on the “pro-organizational” side of UPB. This kind of altruism makes it easy for them to make excuses for their wrongdoings, and no compensation will be taken afterward. As a result, the mediating effect of guilt will be enhanced, hindering the occurrence of individual pro-environmental behaviors. On the contrary, employees with high moral identity have higher moral sensitivity, and they will worry about the “unethical” side of UPB. They are able to behave in a more ethical manner and make compensation for their mistakes. Thus, this study proposes the following hypothesis:

**Hypothesis 5:** Moral identity will moderate the relationship between UPB and employees’ pro-environmental behaviors through guilt, such that the relationship is stronger when moral identity is low than when it is high.

## Materials and methods

### Participants and procedure

In our research, we adopted the research methods used by other scholars, and the data collection was completed by using an online survey questionnaire through the online platform Credamo in China (Gong et al., 2020; Yang et al., 2022). The questionnaire method is generally applied in management research, and this online format allows us to collect a sizable and trustworthy sample conveniently. Moreover, UPB has certain concealability, which is not always perceived by colleagues. Thus, the adoption of anonymous and self-report questionnaires is reasonable to some extent.

We recruited 459 qualified participants through the Credamo platform, whose organizations were mainly located in Guangdong, Zhejiang, and Jiangsu provinces, China, which mainly are covered by industries such as manufacturing, finance, education, and information technology. The participants have satisfied all the following conditions: first, these participants must be full-time employees of the organization; second, the participants must be able to confirm that they had engaged in UPB during their work hours over the past month; finally, the participants need to provide some clear environmental policies and green performance appraisal mechanisms in their organizations. These requirements ensured that selected participants fulfill the goals of the study.

To test the theoretical model, this study used a two-wave survey questionnaire to collect data at two different times, with an interval of 2 months, to reduce the impact of common-method bias (CMB) on the conclusions. In the first wave, after clarifying the principle of voluntary participation, the researcher explained the purpose, process, and anonymity of the survey to ensure that the participants could fill out the questionnaire truthfully. A total of 459 questionnaires were distributed online. After removing samples that had obviously unqualified answers and too short answering time, 399 valid questionnaires were recovered, and each respondent received US$0.42 as a reward. This wave included some core variables such as UPB and guilt, and some basic information such as age, gender, educational background, position, and work tenure. In the second wave, which was two months later, the researchers conducted an online questionnaire survey on 399 qualified subjects from the previous wave and recovered 319 valid questionnaires, and each respondent got US$0.42 as a reward. This wave included some core variables such as moral identity and pro-environmental behavior. The entire survey period was from 24 March to 31 May 2022. Ultimately, 319 valid questionnaires (i.e., a response rate of 86.9%) were recovered for the analysis, and the effective response rate of questionnaire recovery was 69.5%. Among them, 60.5% were women, and 39.5% were men. In terms of age, 1.3% were 18–20 years old, 54.5% were 21–30 years old, 35.1% were 31–40 years old, and 9.1% were 41 years and older. With regard to educational background, 4.1% of the respondents had a high school education or below, 13.2% had a junior college degree, 74.6% had a bachelor’s degree, and 8.1% had a master’s degree or above. Regarding work tenure, 5.7% of the participants worked for less than 1 year, 37.9% for 1–5 years, 37.9% for 6–10 years, and 18.5% for more than 10 years. In terms of work position, 54.9% of the respondents were general employees, 26% were first-line managers, 16.3% were middle managers, and 2.8% were senior managers, as illustrated in Table 1.

**TABLE 1:** Demographics of the samples (*N* = 319).

Variables	Items	Number	Percentage
Gender	Male	193	60.5
	Female	126	39.5
Age	18–20 years	4	1.3
	21–30 years	174	54.5
	31–40 years	112	35.1
	41 or above	29	9.1
Education	High school or below	13	4.1
	Junior college degree	42	13.2
	Bachelor’s degree	238	74.6
	Master’s degree or above	26	8.1
Work tenure	Less than 1 year	18	5.7
	1–5 years	121	37.9
	6–10 years	121	37.9
	More than 10 years	59	18.5
Position	General employees	175	54.9
	First-line managers	83	26
	Middle managers	52	16.3
	Senior managers	9	2.8

### Measures

In this study, all scales used in this research are quoted from authoritative articles and have been published in top journals. Since all the scale items were initially developed in English, two organizational behavior scholars were invited to translate items into Chinese and then back into English. This back-translation method can prevent understanding deviation caused by factors such as cultural differences. The five-point Likert scales ranging from 1 (strongly disagree) to 5 (strongly agree) were used. The factor loadings, CRs, and AVE of each item are shown in Table 2. According to some relevant scholars, if the AVE is less than 0.5 and the CR is higher than 0.6, such a situation is also adequate (Fornell and Larcker, 1981; Lam, 2012).

**TABLE 2:** Measurement validity assessment.

Constructs	Items	Standard factor loadings	Composite reliability (CR)	Average variance extracted (AVE)
Unethical pro-organizational behavior	UPB1	0.711	0.869	0.625
	UPB2	0.761		
	UPB3	0.837		
	UPB4	0.845		
Guilt	G1	0.871	0.915	0.645
	G2	0.829		
	G3	0.826		
	G4	0.768		
	G5	0.802		
	G6	0.714		
Moral identity	MI1	0.616	0.866	0.482
	MI2	0.722		
	MI3	0.725		
	MI4	0.719		
	MI5	0.642		
	MI6	0.660		
	MI7	0.765		
Pro-environmental behavior	PEB1	0.530	0.632	0.301
	PEB2	0.520		
	PEB3	0.570		
	PEB4	0.573		

### Unethical pro-organizational behavior

A scale with six items from Umphress et al. (2010) was used to measure participants’ UPB. The participants were asked to rate the degree of UPB in a self-reported questionnaire, for instance, “If it would help my organization, I would exaggerate the truth about my company’s products or services to customers and clients.” Finally, four items of the initial six-item scale were used to measure the participants’ UPB because these items were appropriate for the research background of participants and most of them are normal office workers with no experience in writing letters of recommendation or charging customers (Tang et al., 2020; Wang Y. et al., 2021). Cronbach’s alpha was 0.865.

### Guilt

A scale with six items from Watson and Clark (1994) was used to measure participants’ guilt (G). The participants were asked to rate the degree of emotional experience (i.e., “Shame,” “blameworthy,” and “dissatisfaction”) when they recall the unethical behavior they did in past weeks that intended to protect their organization (Chen et al., 2022). Cronbach’s alpha was 0.915.

### Moral identity

A scale with seven items from Aquino and Reed (2002) was used to measure participants’ moral identity (MI). They were asked to identify how crucial those moral characteristics (i.e., “caring,” “fairness,” and “justice”) are to them, for instance, “It would make me feel good to be a person who has these characteristics.” Cronbach’s alpha was 0.864.

### Pro-environmental behavior

A scale with four items from Lu et al. (2017) was used to measure participants’ pro-environmental behavior (PEB). A sample item was “Remind and persuade colleagues to protect the environment at the workplace.” Cronbach’s alpha was 0.630. First, this is our first exploratory attempt to link UPB to pro-environmental behavior. Second, Robinson et al. (1991) argued that a value of Cronbach’s alpha is 0.6 could be accepted. Moreover, the values of Cronbach’s alpha for environmental behavior in some articles were 0.630 or 0.690, which are less than the recommended level of 0.700 (Robinson et al., 1991; Harth et al., 2013; Bissing-Olson et al., 2016; Mohammed and Pandhiani, 2017).

### Control variables

The demographic characteristics will exert an influence on the results of the research. Thus, drawing on relevant research experience from the previous study (Kish-Gephart et al., 2010), common demographic variables such as gender, age, educational background, work tenure, and position in an organization were controlled in this study. More precisely, women were coded as 1 and men were coded as 0; age 18–20 was coded as 1, 21–30 as 2, 31–40 as 3, and 41 and above as 4; educational level of high school education or below was coded as 1, a junior college degree as 2, a bachelor’s degree as 3, and a master’s degree or above as 4; work tenure less than 1 year was coded as 1, 1–5 years as 2, 6–10 years as 3, and more than 10 years as 4; and the general employees were coded as 1, first-line managers as 2, middle managers as 3, and senior managers as 4.

## Analysis and results

### Confirmatory factor analysis

To examine the discriminate validity of UPB, guilt, moral identity, and pro-environmental behavior, this study used AMOS 27.0 to conduct confirmatory factor analysis (CFA). Specifically, it includes a four-factor model (theoretical model), a three-factor model, a two-factor model, and a one-factor model. By comparing the CFA analysis results, the four-factor model (χ^2^/df = 2.134, IFI = 0.929, CFI = 0.934, TLI = 0.925, RMSEA = 0.060, and SRMR = 0.059) was better than other alternative models. Furthermore, we added a method factor to the research model to test for CMB (Podsakoff et al., 2003). The quality of the fitted parameters did not have much improvement (△χ^2^/df = −0.12, △CFI = −0.005, △TLI = −0.006, △RMSEA = −0.004, and △SRMR = 0.0017), and the common-method bias in this study did not have a serious impact, as illustrated in Table 3.

**TABLE 3:** Results for confirmatory factor analysis.

Model	χ^2^	df	χ^2^/df	CFI	TLI	RMSEA	SRMR
Four factors	390.463	183	2.134	0.934	0.925	0.060	0.0509
Three factors	560.803	186	3.015	0.881	0.866	0.080	0.0961
Two factors	1,015.519	188	5.402	0.783	0.707	0.118	0.1188
One factor	1,831.384	189	9.690	0.480	0.422	0.165	0.1839
Four factors + CMV	487.443	242	2.014	0.929	0.919	0.056	0.0526

One factor: UPB + GUILT + PEB + MI. Two factors: MI UPB + GUILT + PEB. Three factors: UPB + PEB GUILT MI. Four factors: UPB GUILT PEB MI. Five factors: UPB GUILT PEB MI CMV.

### Descriptive analysis

In order to examine the influence of the common method bias (CMB) which caused by the employees self-reporting questionnaire on the research results, using SPSS 25.0 to check the potential CMB problems. The variance explained by the first factor is 27.4%, which is less than 50% of the total explained variance (62.2%), indicating that there is no serious CMB problem in this research. A test for multicollinearity problems has been conducted. From the VIF values of each variable, it is clear that there is no multicollinearity problem. The means, VIF, standard deviations, and correlations among demographic and four core variables are illustrated in Table 4. SPSS 25.0 was used for this descriptive statistical analysis of the research variables, and the means (standard deviations) of the core variables UPB, guilt, pro-environmental behavior, and moral identity were 2.04 (0.858), 2.13 (0.938), 4.36 (0.447), and 4.03 (0.673), respectively. The results showed that the correlation between UPB and guilt was significantly positive (*r* = 0.444, *p* < 0.01). However, a significant negative correlation exists between UPB and pro-environmental behavior (*r* = −0.318, *p* < 0.01). Guilt was significantly negatively related to pro-environmental behavior (*r* = −0.244, *p* < 0.01). Moral identity was positively related to pro-environmental behavior (*r* = 0.425, *p* < 0.01). These results provide preliminary support for the research hypothesis.

**TABLE 4:** Results of variable descriptive analysis.

Variables	Means	SD	VIF	1	2	3	4	5	6	7	8	9
1. Gender	0.61	0.490	1.067	1								
2. Age	2.52	0.677	2.793	-0.099	1							
3. Education	2.87	0.600	1.145	-0.028	-0.257**	1						
4. Work tenure	2.69	0.835	3.129	-0.205**	0.790**	-0.219**	1					
5. Position	1.67	0.848	1.294	-0.057	0.283**	0.112*	0.394**	1				
6. UPB	2.04	0.858	1.257	0.009	0.062	-0.058	0.008	0.020	1			
7. MI	4.03	0.673	1.059	0.077	0.068	0.018	0.007	0.169**	-0.32	1		
8. G	2.13	0.938	1.269	0.028	-0.019	-0.102	-0.034	-0.020	0.444**	-0.079	1	
9. PEB	4.36	0.447	—	-0.010	0.091	-0.165**	0.090	0.107	-0.318**	0.425**	-0.244**	1

**p < 0.01, *p < 0.05.

### Hypothesis testing

**FIGURE 2:**
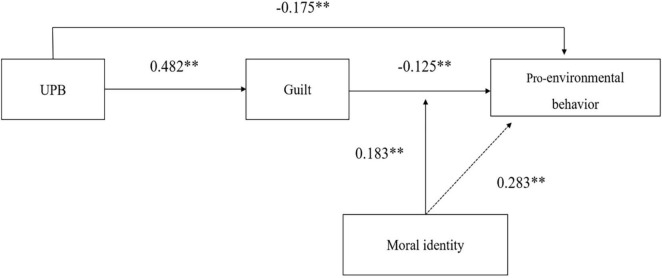
Results of the research model.^**^p < 0.01, *p < 0.05.

This study used hierarchical regression in SPSS 25.0 to test the research hypotheses. The bootstrap method was adopted to test the mediating and moderating effects involved in the research, and the results are illustrated in Figure 2 and Table 5. Hypothesis 1 assumed a direct effect of UPB on guilt. After controlling employees’ gender, age, educational background, work tenure, and position, the regression analysis result was reported, as shown in model 5, which indicated that UPB was positively related to guilt (β = 0.482, *p* < 0.01). Thus, hypothesis 1 was supported. In addition, it can be seen from model 3 that employees’ guilt was negatively related to their pro-environmental behavior (β = −0.125, *p* < 0.01). Thus, hypothesis 2 was supported. The supports of hypotheses 1 and 2 satisfied the two preconditions of the mediation effect.

**TABLE 5:** Result of hierarchical regression analysis.

Variables	PEB						GUILT	
	**M1**	**M2**	**M3**	**M6**	**M7**	**M8**	**M4**	**M5**
Gender	−0.009	−0.010	−0.007	−0.009	−0.035	−0.039	0.017	0.020
Age	0.017	0.048	−0.020	0.043	−0.030	−0.029	0.020	−0.064
Education	−0.133**	−0.146*	−0.156**	−0.157**	−0.132	−0.153**	−0.187*	−0.149
Work tenure	−0.012	−0.035	−0.024	0.037	0.032	0.007	−0.099	−0.035
Position	0.068*	0.074*	0.071*	0.075	0.023	0.041	0.027	0.009
UPB		−0.175**		−0.142**				0.482**
GUILT			−0.125**	−0.068*		−0.109**		
MI					0.283**	0.327**		
GUILT × MI						0.183**		
*R* ^2^	0.044	0.155	0.112	0.171	0.217	0.305	0.015	0.208
Δ*R*^2^	0.028	0.139	0.095	0.153	0.202	0.299	0.001	0.193
*F*	2.860**	9.556**	6.563**	9.187**	14.415**	14.510**	0.983	13.647**

**p < 0.01, *p < 0.05.

Hypothesis 3 assumed that guilt played a mediating role between UPB and pro-environmental behavior. When UPB and guilt were put into the regression model at the same time (model 6), the effect of UPB on pro-environmental behavior was weakened (β = −0.142, *p* < 0.01), and its significance has not disappeared. However, guilt still negatively affected pro-environmental behavior significantly (β = −0.068, *p* < 0.05). In order to further examine the mediating effect of guilt, the bootstrap method was adopted to test the mediating effect. The results showed that the indirect effect of UPB on pro-environmental behaviors *via* guilt was significant (indirect effect = −0.033, SE = 0.017, 95% CI [−0.071, −0.005]). Thus, hypothesis 3 received support.

**FIGURE 3:**
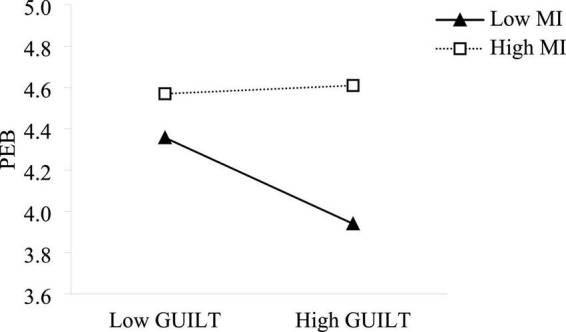
Moderating effect of moral identity on the relationship between guilt and pro-environmental behavior.

Hypothesis 4 assumed that moral identity had a significant moderating effect between guilt and pro-environmental behavior. This study used hierarchical regression to examine the moderating effect of moral identity. In order to avoid the multicollinearity problem caused by the interaction term, centralizing the relevant variables. Model 8 indicated that moral identity was a moderator in the relationships between guilt and pro-environmental behavior (β = 0.183, *p* < 0.01), which indicates the interaction term had a significant effect on pro-environmental behavior. In order to further test the moderating effect, this study drew a chart of the moderating effect at different levels, as illustrated in Figure 3. When employees had low levels of moral identity, guilt had a stronger negative impact on pro-environmental behaviors. The negative impact of guilt on pro-environmental behaviors is weaker when employees have high levels of moral identity. Thus, hypothesis 4 was supported.

Hypothesis 5 proposed that the indirect effect of UPB on pro-environmental behavior *via* guilt could be moderated by moral identity. In this study, the bootstrap method from PROCESS was adopted to test the moderated mediation effect, and the result is shown in Table 6. When employees had low levels of moral identity (−1 SD), the indirect effect of UPB on pro-environmental behavior *via* guilt was −0.080, 95% CI [−0.1443, −0.0297]. For employees with high moral identity (+1 SD), the indirect effect of UPB on pro-environmental behavior *via* guilt was 0.039, 95% CI [0.0008, 0.0838]. This finding suggested that under the condition of different levels of moral identity, the effect of UPB on pro-environmental behavior through guilt was significantly different. Thus, hypothesis 5 was supported.

**TABLE 6:** Results of conditional indirect effects.

Moderator variable	GUILT		
	**Boot indirect effect**	**Boot SE**	**95% CI**
High moral identity (1 + SD)	0.039	0.021	[0.0008, 0.0838]
Low moral identity (1 − SD)	−0.080	0.029	[−0.1443, −0.0297]

## Discussion

Based on the conservation of resources theory, a research model with guilt as the mediating variable and moral identity as the moderating variable was constructed. This research explores the impact mechanism and boundary conditions of UPB on individual pro-environmental behaviors from the perspective of employees. The results based on 319 Chinese employees’ data show that (1) guilt plays a mediating role between UPB and pro-environmental behaviors; (2) UPB can induce employees’ feelings of guilt and then guilt will affect employees’ pro-environmental behaviors; and (3) moral identity moderates the relationship between guilt and pro-environmental behaviors, as well as the indirect influence of UPB on pro-environmental behaviors through guilt. Next, we discuss the theoretical and practical implications, limitations of the current work, and future research directions.

### Theoretical implications

First, this study expands the empirical research of the influence mechanism of UPB, and the aftereffects of UPB on employees’ attitude and work behavior has been the focus of organizational behavior (Liu et al., 2021; Wang Y. et al., 2021). However, a retrospection of the previous literature indicated that most of the current research studies on UPB explored the antecedents and formation mechanism of this behavior from the perspective of organization, leadership, or individual factors (e.g., ethical climate, leadership style, and perceived organizational support), but the research on the consequences of UPB was often ignored by scholars (Chen et al., 2016; Babalola et al., 2021; Sheedy et al., 2021). Moreover, even if there are some studies on the outcome of UPB that were usually based on the perspective of moral compensation, individuals would take compensatory behaviors after realizing their wrongdoings or unethical behavior (Wang and Xiao, 2018; Wang Y. et al., 2021). These studies lacked new research perspectives and theoretical applications. Drawing on the conservation of resources (COR) theory, our study offered a different view of such conventional thinking and indicated that moral compensation effects did not necessarily occur because the mental resources losses caused by UPB prevented individuals from taking compensatory behaviors. Although some of the existing literature pointed out the complex links between ethical or unethical behavior in business and pro-environmental behavior (Delmas and Burbano, 2011; Gamma et al., 2018; Larue et al., 2022), limited research has empirically examined the effect of UPB on pro-environmental behaviors in the workplace. This study empirically tested the relationship between UPB and pro-environmental behaviors and enriched the literature of green behavior in the workplace by illustrating employees’ UPB outcomes, which could deepen the knowledge and understanding of the negative effects of UPB.

Second, this study revealed the underlying psychological mechanisms of individuals’ UPB influence on their pro-environmental behaviors from a resource conservation perspective, and the “black box” between the two was opened by introducing the mediating role of guilt. The existing studies on the relationship between environmental behavior and ethical factors in work were usually based on a social exchange theory perspective, and mediating roles were explored in terms of hypocrisy, job satisfaction, and other factors (Paillé et al., 2015; Gamma et al., 2018). By comparison, based on the COR theory and the principle of the primacy of resource loss, our study focused on the role of guilt in the depletion of psychological resources and pointed out the negative effect of UPB on employees’ pro-environmental behaviors was achieved through the emotional process of exhaustion caused by guilt. Moreover, some studies suggested that UPB was highly likely to induce moral emotions such as guilt (Umphress and Bingham, 2011), and guilt often stems from individuals’ negative self-evaluation when they perceived the behavior violation of social ethics (Tang et al., 2020), as well as our findings reconfirmed this view. In addition, in the previous green literature, most studies suggested that there was a positive relationship between guilt and pro-environmental behaviors (Onwezen et al., 2014). This study broke previous research conventions and provided a new perspective on understanding the relationship between guilt and pro-environmental behavior.

Third, our research focused on the level of morality with individual differences and explored the boundary conditions of the effect of guilt on employee psychological states and behavior and the indirect effect of UPB on pro-environmental behavior through guilt by incorporating the moderator of moral identity. Some previous studies have shown that the level of moral identity would influence individuals’ subsequent mental states and behavioral decisions when they were confronted with unethical events (Greenbaum et al., 2014; Joosten et al., 2014). Our study obtained a consistent conclusion that moral identity can effectively moderate the compensatory behavior of individuals. The results showed that high moral identity can attenuate the negative impact of guilt on pro-environmental behavior and the indirect influence of UPB on pro-environmental behavior *via* guilt. Individuals with a high level of moral identity placed a prominent value on the significance of moral character and were more likely to adopt compensatory behaviors (e.g., pro-environmental behaviors) when they recognized their wrongdoings. Conversely, individuals with low moral identity did not tend to fix mistakes after engaging in UPB, and the possibility of generating compensatory behavior was low. Thus, this study enriched the boundary conditions of UPB mechanisms.

### Practical implications

First, our findings indicate that the moral compensation effect does not necessarily occur. Conversely, the depletion of emotional resources generated by UPB may contribute to further the risk of moral slippage among employees, and individuals will devote fewer resources to pro-social or complementary behaviors. So, even though UPB has a pro-organizational character and can bring temporary benefits to the organization, managers should recognize that UPB is not conducive to pro-social behavior (e.g., pro-environmental behavior) and the development of a sense of social responsibility among employees, which would be detrimental to the long-term development of the organization. So, it is suggested that managers should be aware of the employees’ invisible behaviors that are related to ethics and help employees identify unethical elements in their daily work behaviors, and this could enable managers to prevent the occurrence of UPB in time. Therefore, organizational managers should regularly conduct moral training activities to help employees establish the appropriate professional ethics and guide employees establish long-term goals to avoid short-term unethical behaviors such as UPB. At the same time, managers should keep the core values of the organization consistent with the core social values and ethics advocated by the state, which could foster a sense of social responsibility among employees to inhibit UPB.

Second, we investigated the mediating mechanism of guilt in the process of UPB affecting pro-environmental behavior. The guilt-induced depletion of individuals’ emotional resources could lead to a decrease in their pro-environmental behavior. We advocate that organizations should adhere to an “employee-foremost” management philosophy, and managers should not only provide employees with the job skill training but also give them psychological guidance when they are confronted with negative moral emotions. Organizations can help employees relieve negative emotions in a timely and effective manner by setting up psychological counseling chambers, mental anti-stress training, and emotional release rooms, and these measures can supplement employees’ psychological resources and reduce the individual’s emotional internal conflict.

Finally, consistent with previous studies, employees with a higher level of moral identity not only had higher moral standards for themselves but also were more prone to take compensatory actions to compensate for their wrongdoings after discovering the unethical nature of UPB. This finding has several practical implications and means that there were some intervention strategies that organizations can devise to improve the overall moral values of their employees. When organizational recruiting, managers should reasonably assess the candidates’ level of intrinsic ethical standards during the interview process, identifying and hiring individuals who value morality in self-schema. Moreover, organizations should conduct training on correct ethical values and behavioral norms for staff and appraise the daily ethical behavior of trainees, which could build a highly ethical workforce.

### Limitations and future directions

Although this study proposed some original views on the impact mechanism of UPB, there are still some shortcomings in the process: First, all data were collected in two waves, and this research adopted the method of self-assessment, which had a large subjective component and may result in CMB. These participants might have evaluated themselves as too high or too low. Therefore, in future research, qualitative methods such as depth interviews should be adopted to obtain a more comprehensive understanding of the participants’ conditions. Second, there was a lack of comparative analysis of industry samples, so in further research, the number of industries should be appropriately increased to make the sample more representative. Third, this study did not examine the impact of emotional exhaustion on employees’ subsequent behavior. The degree of individual emotional exhaustion can more comprehensively explain the effects of UPB on employees’ psychological state and work behavior. Furthermore, although the adoption of anonymous methods and online platforms to distribute questionnaires can alleviate the participants’ concerns about their privacy, there is still a problem of the thoughtless consideration of potential control variables. This study only controlled common demographic variables such as gender, age, and position based on past experience. Future studies can use field trials to collect data to retest the theoretical model in this study. Finally, future research can consider pro-social behavior as a way to supplement resources. If pro-social behavior is viewed as a way to supplement resources, then this will also bring us the new implication of management practice. Environmentally friendly behaviors can further compensate for their lack of psychological resources, and pro-social behaviors such as pro-environmental and organizational citizenship behaviors promoted by managers within the organization can mitigate the effects of negative emotions on employees.

## Conclusion

Although there were many studies on unethical pro-organization behavior in the past, most of those focused on the inducing factors and formation mechanism of the behavior, and had a lack of attention to its potential subsequent impact on individuals. In this research, we aimed to explore the mechanisms and boundary conditions under which UPB can influence individuals’ pro-environmental behavior. The study found that when individuals engage in UPB, they would feel guilty, which further reduced the possibility of individuals’ participation in pro-environmental behavior. Furthermore, moral identity can moderate this indirect relationship. This study constructed a research model based on the conservation of resources theory and tried to explore the relationship between UPB and employees’ pro-environmental behavior, as well as clarified the reasons why this behavior can weaken employees’ pro-environmental behavior. Moreover, this study also tried to understand the influence of UPB on individuals’ cognition and behavior and further enriched and expanded the theoretical system of UPB, which provided practical implications for managers on how to control UPB.

## Data availability statement

The raw data supporting the conclusions of this article will be made available by the authors, without undue reservation.

## Ethics statement

The studies involving human participants were reviewed and approved by the Ethics Review Committee of the School of Management, Harbin Institute of Technology. Written informed consent for participation was not required for this study in accordance with the national legislation and the institutional requirements.

## Author contributions

MZ and SQ designed the study and constructed the theoretical model. MZ analyzed the data and wrote the manuscript. SQ reviewed the manuscript. Both authors approved the final version of the manuscript.
